# Utilization of vinasses as soil amendment: consequences and perspectives

**DOI:** 10.1186/s40064-016-2410-3

**Published:** 2016-07-07

**Authors:** R. G. Moran-Salazar, A. L. Sanchez-Lizarraga, J. Rodriguez-Campos, G. Davila-Vazquez, E. N. Marino-Marmolejo, L. Dendooven, S. M. Contreras-Ramos

**Affiliations:** Unidad de Tecnología Ambiental, Centro de Investigación y Asistencia en Tecnología y Diseño del Estado de Jalisco A.C. (CIATEJ), CP 44270 Guadalajara, Jalisco Mexico; Unidad de Servicios Analíticos y Metrológicos, Centro de Investigación y Asistencia en Tecnología y Diseño del Estado de Jalisco A.C. (CIATEJ), CP 44270 Guadalajara, Jalisco Mexico; Unidad de Biotecnología Médica y Farmacéutica, Centro de Investigación y Asistencia en Tecnología y Diseño del Estado de Jalisco A.C. (CIATEJ), CP 44270 Guadalajara, Jalisco Mexico; Laboratorio de Ecología de Suelos, ABACUS, Cinvestav, Mexico

**Keywords:** Agave, Greenhouse gases, Mezcal, Sugar beet, Sugarcane, Tequila

## Abstract

Vinasses are a residual liquid generated after the production of beverages, such as mezcal and tequila, from agave (*Agave* L.), sugarcane (*Saccharum officinarum* L.*)* or sugar beet (*Beta vulgaris* L.). These effluents have specific characteristics such as an acidic pH (from 3.9 to 5.1), a high chemical oxygen demand (50,000–95,000 mg L^−1^) and biological oxygen demand content (18,900–78,300 mg L^−1^), a high total solids content (79,000 and 37,500 mg L^−1^), high total volatile solids 79,000 and 82,222 mg L^−1^, and K^+^ (10–345 g L^−1^) content. Vinasses are most commonly discarded onto soil. Irrigation of soil with vinasses, however, may induce physical, chemical and biochemical changes and affect crop yields. Emission of greenhouse gases (GHG), such as carbon dioxide, nitrous oxide and methane, might increase from soils irrigated with vinasses. An estimation of GHG emission from soil irrigated with vinasses is given and discussed in this review.

## Background

Vinasses are a residual liquid generated during ethanol production from sugarcane (*Saccharum officinarum* L.*)* or sugar beet (*Beta vulgaris* ssp. *vulgaris* var. *altissima Döll*), or distillation of beverages, such as mezcal and tequila. They are characterized by a dark color, acid pH, a high electrical conductivity (EC), large amounts of organic matter, and high concentrations of suspended and volatile solids and occasionally contain heavy metals (USEPA 2004; CETESB 2006). Vinasses are not hazardous waste (EPA 2016), but they are considered a complex wastewater due to their composition. Commonly, vinasses are used as a fertilizer due to their high plant nutrient content, mainly calcium (Ca) and potassium (K), and their high organic material content, which could be mineralized and alter the nitrogen and carbon cycles improving greenhouse gases emissions such as CO_2_, CH_4_ and N_2_O with high moisture conditions. However, their discharge in the environment can contaminate soil and groundwater as they often contain salts, metals and dissolved solids (Prasad et al. 2008).

## Type of vinasses: origin and production

The vinasses are generated mainly during distillation. According to the FAO (2015), the largest producer of sugarcane, ethanol and sucrose in the world is Brazil followed by India, China, Pakistan, Thailand, Mexico, Colombia, Australia, South Africa and Cuba (Table 1) (Bassanta et al. 2003). It has been estimated that between 10 and 15 L of sugarcane vinasses (SC) are generated per liter of ethanol produced (Cavalett et al. 2012). According to the latest data from FAO (2015), ethanol production from sugarcane in the world was 1.4 × 10^11^ L ethanol in 2013, which could have generated 1.4–2.1 × 10^12^ L sugarcane vinasses.

**Table 1:** Origin and production of vinasses

Type of vinasses	Raw material	Origin	Estimate generation	Producers in the world*	References
Sugarcane	*Saccharum officinarum*	In distillation of ethanol	In Brazilian: 10–15 L per liter of ethanol	Brazil, India, China, Pakistan, Thailand, Mexico, Colombia, Australia, South Africa and Cuba	Bassanta et al. (2003)
Sugar beet	*Beta vulgaris*	In distillation of ethanol	9–14 L per liter of ethanol	Germany, France, Poland, UK, Canada, US, South Korea, Japan and India	Prasad et al. (2008),
Mezcal	*Agave salmiana*, *Agave angustifolia, Agave potatorum*	In the distillation and rectification stage	From 8 to 15 L per liter of mezcal	Mexico	Robles-González et al. (2012)
Tequila	*Agave tequilana Weber* var. Azul	In the distillation	10 L per 1 L of tequila	Mexico	Méndez-Acosta et al. (2010)

* FAO (2015)

Another source of vinasses is sugar beet, which is used to produce sucrose and ethanol (Prasad et al. 2008). It grows mainly in Europe (Germany, France, Poland and UK), North America (Canada and US), Asia (South Korea, Japan) and India (Joersbo 2007). The production of beet vinasses in an ethanol factory ranges from 9 to 14 L vinasses per L ethanol obtained (Jiménez et al. 2003). In 2013, the production of ethanol from sugar beet was 2.3 × 10^10^ L around the world, which could have generated from 2 to 3.2 × 10^11^ L vinasses (FAO 2015).

Mezcal is a Mexican traditional distilled beverage produced by fermenting the juices of cooked agave cores. Mezcal is produced from various species of Agave, mainly *Agave salmiana*, *A. angustifolia* and *A. potatorum*. According to “*Consejo Regulador del Mezcal* (*Mezcal Regulatory Council*)” (CRM) (CRM 2015), the annual production of mezcal in Mexico is 4.2 × 10^6^ L year^−1^ and it is assumed that for each liter of mezcal produced between 8 and 15 L of vinasses are generated (Robles-González et al. 2012). As such, from 1.6 to 2.3 × 10^9^ L vinasses were produced from mezcal production in 2013.

Another Mexican distilled drink is tequila. It is obtained from the *A. tequilana* weber var. azul. This is the only variety of agave permitted to apply the “appellation of origin” for tequila. The tequila production was 2.4 × 10^8^ L in 2014 (CRT 2015). It has been reported that the tequila industry generates between 7 and 10 L of tequila vinasses L^−1^ tequila produced. As such, between 1.7 and 2.4 × 10^9^ L vinasses were generated from tequila production in 2014 (Méndez-Acosta et al. 2010).

Mezcal and tequila vinasses are generated only in Mexico, but they present the same problems of treatment, management and final disposition as other kind of vinasses. The average volume of vinasses generated by processing sugarcane, production of alcohol and distilled beverage ranges between 7 and 15 L^−1^ of final product. The vinasses share some characteristics but also variations in physicochemical parameters, which are described below.

## Characteristics of different type of vinasses

The characteristics of vinasses depend mainly on the raw material used, but all of them share some similar properties, such as an acidic pH (from 3.9 to 5.1), a high chemical oxygen demand **(**COD) (50,000–95,000 mg L^−1^) and biological oxygen demand (BOD) content (18,900–78,300 mg L^−1^) (Table 2). The mezcal and tequila vinasses have a high total solids content (79,000 and 37,500 mg L^−1^ respectively). The total volatile solids content in sugar beet and sugarcane vinasses are high, i.e. 79,000 and 82,222 mg L^−1^, respectively (Table 2). If they are applied to soil, the amount of the organic material, nitrate (NO_3_^−^) and ammonium (NH_4_^+^), potassium (K^+^), calcium (Ca^2+^), magnesium (Mg^2+^), sodium (Na^+^) and metals will increase in soil and when leached out might contaminate groundwater. Brito et al. (2009) reported that the addition of organic matter to soil increases oxygen consumption and creates anaerobic microsites, leading to a decrease in the redox potential of the soil. This promotes eutrophication and undesirable changes in ecosystems and their functioning. Eutrophication is more knew in aquatic systems, this is the process through which lakes, streams, or bays become overloaded with excess of nutrient such as nitrogen and phosphorus. When the aquatic life die, microorganisms feed of the remains as part of the decomposition process and consequently consume the available oxygen in the water. This leaves little oxygen for fish and other aquatic animals, resulting in the suffocation of aquatic life. Eutrophication can also occur in soils (SSSA 2016). Excess phosphorus and nitrogen content in vinasses could cause eutrophication in aquifers and in soils when they are irrigated in high doses, flooding the pores, decreasing the aeration, promoting the soil saturation with inorganic P, salts, ions and other compounds generated during organic matter decomposition.

**Table 2:** Physicochemical characteristics of different vinasses and American environmental regulations for irrigation

Parameter	Vinasses				
	Sugarcane	Beet	Mezcal	Tequila	USEPA (2004)*
pH	3.8–4.7^a^	4.3–5.35^b^	3.6–3.8^a^	3.4–4.5^bl^	6
Electrical Conductivity (EC) (mS cm^−1^)	16^c^	35–40^dg^	2.6–4.2^a^	0.00195^f^	NR
Phosphates (PO_4_^3−^) (mg L^−1^)	20–233^a^	120^g^	290–1705^a^	100–700^l^	NR
Total Phosphorus (TP) (mg L^−1^)	1–190^b^	160–163^b^	NR	41^b^	NR
Total Organic Carbon (TOC) (g L^−1^)	26–32^a^	196–592^dh^	NR	16.8^m^	NR
Total Nitrogen (TN) (mg L^−1^)	975^a^	1800–4750^b^	660^a^	20–50^l^	NR
Chemical Oxygen Demand (COD) (g L^−1^)	59–80.5^a^	55.5–91.1^b^	56.2–123^a^	55.2–66.3^b^	NR
Biological Oxygen Demand (BOD) (g L^−1^)	31.5–75^a^	27.5–44.9^b^	NR	20.6^b^	45
Total solids (g L^−1^)	63–69^a^	109^g^	26–95^a^	25–50^j^	N.R
Total Suspended solids (g L^−1^)	3–11^a^	3.6^g^	3.1–8.4^a^	2–8^l^	45
Volatile Suspended solids (g L^−1^)	2.5–9^a^	2.5^g^	1.1–6.8^a^	1.9–7.5^l^	N.R
Total Volatile solids (g L^−1^)	82	NR	NR	NR	N.R
Cadmium (Cd) (mg L^−1^)	0.04–1.36^b^	<0.1^h^	NR	0.01–0.2^b^	0.01–0.05
Copper (Cu) (mg L^−1^)	NR	2.1–5^b^	NR	0.36–4^b^	0.2–5
Chromium (Cr) (mg L^−1^)	NR	<0.01^h^	NR	NR	0.1–1
Mercury (Hg) (mg L^−1^)	NR	<0.001^i^	NR	NR	0.002
Lead (Pb) (mg L^−1^)	0.02–0.48^b^	<5^b^	NR	0.065–0.5^b^	5–10
Nickel (Ni) (mg L^−1^)	NR	<0.1^h^	NR	<0.02^l^	0.2–2.0
Zinc (Zn) (mg L^−1^)	15^a^	11^h^	NR	<1^l^	2–10
Iron (Fe) (mg L^−1^)	12.8–203^ba^	203–226^b^	NR	35.2–45^b^	5–20
Phenols (mg L^−1^)	34^b^	450^b^	478–542^a^	44–81^b^	NR
Potassium (K) (g L^−1^)	30^a^	10–10.03^b^	NR	240–345^b^	NR
Density (g cm^−1^)	NR	1.26^h^	NR	NR	NR

* Permissible limits for application to soil; ^a^ Robles-González et al., (2012), ^b^ España-Gamboa et al. (2011), ^c^ Bautista-Zúñiga et al. (1998), ^d^ Núñez-Zofío et al. (2013), ^e^ Conde-Bueno et al. (2009); ^f^ Iñiguez et al. (2005); ^g^ Jiménez et al. (2003); ^h^ Tejada et al. (2009); ^i^ Tejada et al. (2007); ^j^ Santos et al. (2014); ^k^ Vlyssides et al. (2010); ^l^ López-López et al. (2010); ^m^ Personal communication; *NR* not reported

The mineralization of soil organic matter or the applied organic waste will alter the nitrogen and carbon cycles (Buschiazzo et al. 2004). During organic material decomposition under aerobic conditions, C substrate can be transformed to bicarbonate (HCO_3_^−^), carbonates (CO_3_^2−^) and carbon dioxide (CO_2_), while under anaerobic conditions, acetates are formed and C is converted to methane (CH_4_) and carbon dioxide (CO_2_) (Thangarajan et al. 2013).

Organic nitrogen from organic matter is mineralized to ammonia (NH_3_) and under aerobic conditions ammonium (NH_4_^+^) is oxidized to nitrite (NO_2_^−^) and nitrate (NO_3_^−^) while N_2_O is formed as a by-product. Under anaerobic conditions, NO_3_^−^ is reduced to NO_2_^−^, nitric oxide (NO), nitrous oxide (N_2_O) and dinitrogen (N_2_) (Wrage et al. 2001). The mineralization of organic N depends on various factors, such as soil type, temperature, water content, aeration, nature from organic material and the C/N ratio. Soils applied with a high C/N ratio (>19) may be characterized by a low N mineralization or immobilization of N and soils with a low C/N ratio (≤14) by a high N mineralization or slow N immobilization (Bengtsson et al. 2003; da Silva et al. 2012). The total nitrogen (N) content of vinasses ranges from 0.974 to 4.75 g L^−1^ while the carbon content ranges from 26 to 592 g L^−1^ so it has a C/N ratio >27 (Table 2). The N content of vinasses is low so that the C/N ratio is high, which could suggests nitrogen immobilization by vinasse addition. This had been reported by Parnaudeau et al. (2008) with irrigation of crops of sugarcane vinasses. They observed a nitrogen immobilization induced at the start of an experiment in laboratory conditions. However, there are not reports of nitrogen mineralization or immobilization in field conditions.

The total phosphorus (P) content of sugarcane and sugar beet vinasses is high compared to other vinasses (Table 2). The USDA (2014) classifies soils according to phosphorus content as *very low* (0–5 mg P kg^−1^), *low* (4–15 mg P kg^−1^), *medium* (11–24 mg P kg^−1^), *high* (17–30 mg P kg^−1^), and *very high* (>30 mg P kg^−1^). For instance, crops with highest production in the world (wheat, rice, corn, sorghum, potato) require a recommended dose of phosphorus of 6.5, 6.5, 9.9, 13.2 and 17.2 kg P ha^−1^ respectively (FAO 2000, 2015). If we add the dose recommended by Goncalves de Oliveira et al. (2013) of 200 m^3^ ha^−1^ of vinasse to agricultural field with that crops, would add 38, 20.6 and 8.2 kg P ha^−1^ with sugarcane, sugar beet and tequila vinasses respectively. This could exceed the phosphorus required by the plant and it could be mineralized or lixiviated into the soil depending on pH, the type of soil (sand, sandy-loamy, clay, etc.), crop, season, mineralogical characteristics, etc. (FAO 2000).

An excess of inorganic P may induce saturate soil with inorganic P, which could filter down to the groundwater, induce micronutrient deficiency, such as iron and zinc (Osman 2013). Also it can alter the function of the arbuscular mycorrhizal fungi (AMF) and their spore densities in soil. Arbuscular mycorrhizal fungi are beneficial organisms for soil and plants (Xu et al. 2014). It has been reported that application of alcohol vinasse reduced the length and amount of the alive and active external mycelium in AMF (Kabir et al. 1998), sugarcane vinasses decreased the glomalin content in soil with AMF (Velásquez-Pomar and Sánchez de Prager 2011). However there are few information regarding how the vinasses affect AMF population or if they survive after vinasses irrigation.

The K^+^ is an essential micronutrient for plants at low concentrations (16–450 kg ha^−1^) which depends on physiologic stages of the plant (Roy et al. 2006). Some crops require or are tolerant to a high amount (270–300 kg ha^−1^) of K^+^ such as alfalfa (Medicago sativa), corn (Zea maize), grain Sorghum (Sorghum bicolor) (Roy et al. 2006). So, the vinasses could be irrigated in these kind of crops or those where the plants use this cation for grow or maintaining it in a high demand.

In the vinasses the K^+^ content is high (>10,000 mg L^−1^) in sugarcane, sugar beet and mezcal. Qiu et al. (2014) reported that a high K^+^ application rate (186.7 kg K^+^ ha^−1^) to maize reduced the grain growth and consequently reduced yields. Other authors reported positive effect in the plants (pea and sunflower) in yield, biomass and foliar area, but only at low rate (2.5 %) application of sugar beet vinasses (Algur and Kadioglu 1992). In addition, Poz-Gonzalo et al. (2006) reported that some areas in Brazil, have shown serious problems with K^+^ lixiviation, as a 2006 consequence of high vinasses irrigation rates in the last decade. Recently, Ortegon et al. (2016) reported an increase (≈5 % of TSD) in main ions into groundwater under sites irrigated with sugarcane vinasses in Colombia during last decade.

The irrigation of vinasse has as a consequence the accumulation of salts due to high concentrations of Na^+^, Ca^++^, Mg^++^, K^+^ among other cations, the high irrigation rates, frequency, and the intrinsic characteristic of each site, which in conjunct determine the toxicity of vinasses by salt accumulation. Besides it is well know that soil salinization has impacts such as increase of osmotic potential, and destruction of the soil structure by dispersing the soil particles and clogging up pores (Fuess and Garcia 2014).

In general, the heavy metal content in all vinasses is lower than limits established by USEPA for irrigation of soil with wastewaters (USEPA 2004). The content of total N, total P, COD, phenols and K^+^ are not regulated for soil irrigation of vinasses in many countries and by USEPA (2004), which could cause several environmental issues.

Other main problem of vinasses is its usual dark brown color and the presence of high amount of polyphenolic compounds (34–542 mg L^−1^) (Table 2), such as tannic acid, humic acid, carbohydrates and furfurals from acid hydrolysis (Pant and Adholeya 2007). Phenolic compounds can have a phytotoxic effect on plant tissues during germination and seedling development (Casa et al. 2003). Additionally, phenolic compounds and melanoidins can repress biological treatments of vinasses and inhibit the activity of microorganisms in soil and water bodies (Parnaudeau et al. 2008). This will be discussed in other section of this manuscript.

Different technologies, such as aerobic and anaerobic treatments, adsorption, coagulation-flocculation, ozonisation, electrochemical oxidation and electro-coagulation, have been explored to reduce the contaminants in vinasses. All of them have proven to be appropriate as pre-treatments and post-treatments for the reduction of color and organic matter (from 52 to 92 % measured as COD) (Robles-González et al. 2012).

Despite the wide range of technologies available to treat vinasses, the lack of regulations from environmental authorities makes the application of vinasses to soil or water bodies, such as rivers or streams, the most common way of discharging them (Moraes et al. 2014). Brazil’s legislation allows the irrigation of agricultural fields with vinasses with the only restriction that <185 kg potassium oxide (K_2_O) is applied per year (CETESB 2006). However, these regulations do not establish limits for other possible contaminants.

## Application of vinasses to soil and crops

**Fig. 1:**
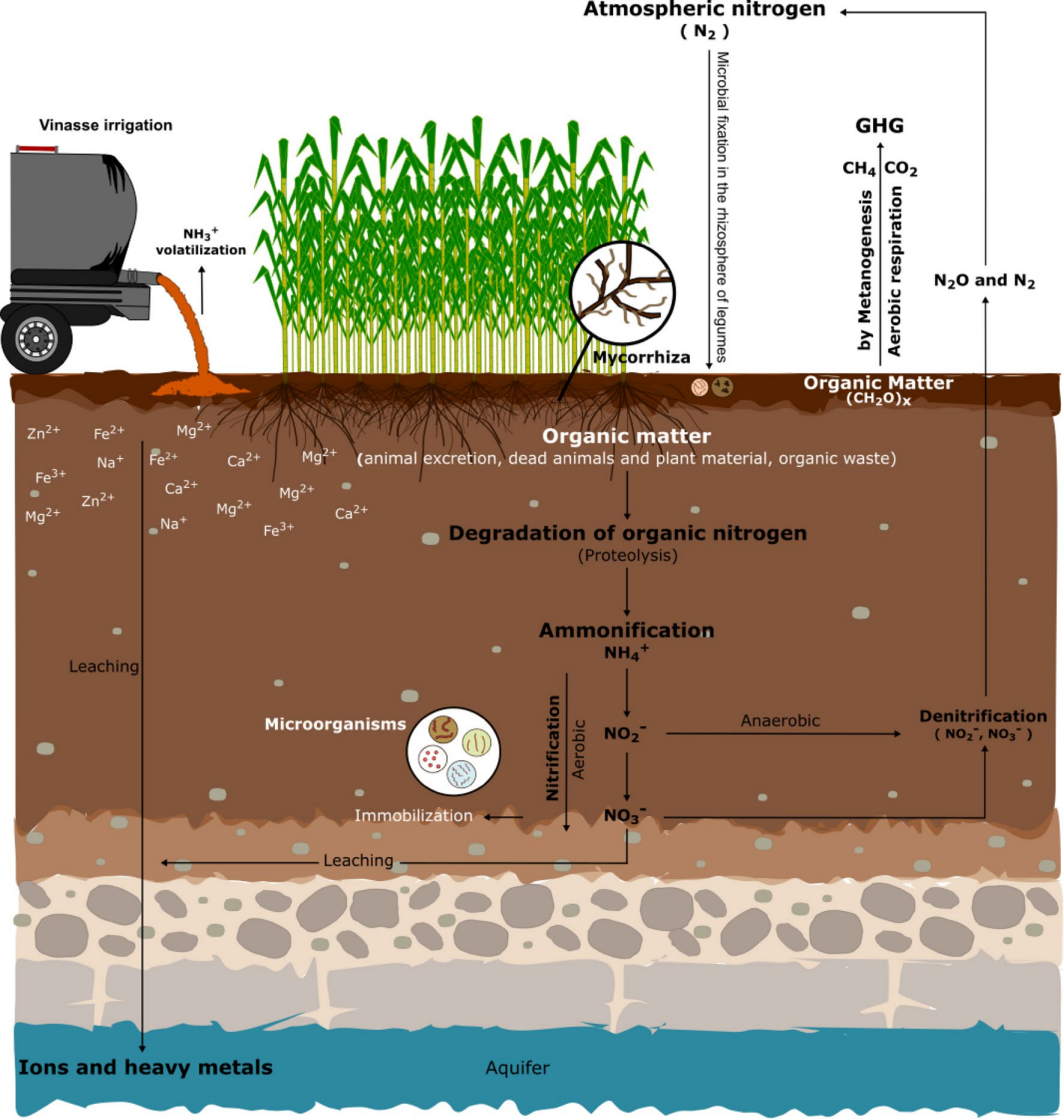
Schematic representation of some processes that might be affected by the addition of vinasses to soil

Irrigation with vinasses to soil induces physical, chemical and biochemical changes in soil properties. The first change is noted with deposition of organic material on topsoil and hardening of this, some authors have reported compaction and decrease of permeability between other effects either positive or negative. According with several authors (Madejón et al. 2001; Tejada and Gonzalez 2006; Bermejo 2010; Moraes et al. 2014) the application of vinasse to soil has different effects due to factors such as the amount applied to the soil that usually is very high (i.e. 200, 300 m^3^ ha), soil type (sandy, clay or loamy or their combinations), the chemical composition of soil (kind of mineralogy), type and age of the crop at moment to irrigation, season (dry or rainy), etc. The Fig. 1 gives a schematic view of possible changes or process that could be altered with vinasses irrigation; some of them are based in effects reported for different types of vinasses, which are discussed in next subsections.

### Sugarcane vinasses

Sugarcane vinasses have been used as fertilizer for sugarcane crops as they are rich in organic material and plant nutrients, i.e. (K, N, and P). Moraes et al. (2014) mentioned that the environmental impact of sugarcane vinasse on soil, however, have not been determined properly. They showed that addition of sugarcane vinasses increased NO_3_^−^ content in soil, which was leached out to the ground water. Others authors mentioned that possible effects of sugarcane vinasses are depending on the quantity and composition of the vinasse applied, soil type, relief, crop type and the economic conditions involved in the process (Aparecida-Christofoletti et al. 2013).

Studies done by Laime et al. (2011) found that disposal of sugarcane vinasse to soil had beneficial effects on crops and some physicochemical characteristics, such as an increase in moisture retention, porosity, K^+^, EC, and biological activity. Ribeiro et al. (2010) detected an increase in the leaching of lead when sugarcane vinasses were applied to soil, particularly the soluble organic compounds. They formed a soluble complex of organic matter-lead, which might be leached out. Parnaudeau et al. (2008) suggested that the irrigation of sugarcane crop with vinasses induced N immobilization at the beginning of an incubation experiment in the laboratory. However, they did not mention how that would affect mineral N in a field experiment. In general, there is little information about N mineralization when sugarcane vinasses are applied to soil or N availability for plants.

### Sugar beet vinasses (SB)

Some authors reported that the application of sugar beet vinasses to soil decreased bulk density as a result of dilution of the deep soil mineral fraction (Madrid and Díaz-Barrientos 1998; Tejada et al. 2007). All vinasses have high content of monovalent cations, which can cause dispersion of organic matter and clay particles, breaking of aggregates and soil structure. The dispersed clay particles can block pores, cause hardening of the soil upon drying, decrease water infiltration and permeability, and as consequence reduce plant growth (Mavi et al. 2012). Other authors reported that soil structural stability; microbial biomass, soil respiration, and dehydrogenase, urease and phosphatase activity was reduced (Tejada et al. 2007). In addition, an increase in monovalent cation content and fulvic acids has been reported, which indicates mineralization of organic matter (Tejada et al. 2007).

Madejón et al. (2001) indicated that sugar beet vinasses have a great potential in agriculture because of their high organic matter, N and K content. However, was reduced their potential use by a high Na^+^ content (21 g L^−1^), which is responsible for increased soil erosion and a decrease in microbial biomass and crop productivity. Contrarily, Gemtos et al. (1999) reported an increase in K^+^ content in the soil, which was beneficial for durum wheat (*Triticum durum* L.). The yield of durum wheat increased with 32 or 46 % when sugar beet vinasses were applied to soil at 3500 or 7000 kg/ha, respectively. They suggested that applications every 3–4 years could avoid any adverse effects on soil and cultivated crops. Martín-Olmedo et al. (1999) observed that the application of sugar beet vinasses to soil for 3 years increased the N mineralization potential. They stated that mineral N (NO_3_^−^ and NH_4_^+^) might be immobilized in organic form, which is available to plants only slowly. However, an excess of NO_3_^−^ might filter thought soil and contaminate aquifers.

Other authors have suggested that using sugar beet vinasses in a composting process with other solid wastes could solve some of the above mentioned problems (Robles-González et al. 2012). Although, the composting of vinasses with agricultural wastes has been shown to be suitable, the compost from vinasses should be stable and mature before its application to soil. The high salt concentration of the sugar beet vinasses will be diluted after composting, but not eliminated so application rates of the compost with vinasses should be controlled carefully.

### Mezcal and tequila vinasses

The application of mezcal and tequila vinasses to soil is a common practice in Mexico. However, there are not reports on possible effects on soil by application of vinasses. It can be assumed that soil quality will be affected negatively as has been reported for other vinasses, although some positive effects have been reported too, such as with sugarcane vinasses. The main negative effect of added vinasses on soil is the accumulation of salts and cations (K^+^, Na^+^, Mg^++^) on soils, which increased salinity and sodicity (Tejada et al. 2009). Also, excess organic material application can reduce porosity, promoting anaerobic conditions and phytotoxicity to crops due to accumulation of organic compounds, such as acetic acid, lactic acid and glycerol (Yavuz 2007). Mineralization of the organic N can lead to excess mineral N in soil, which can be leached out (mostly NO_3_^−^) to rivers and aquifers. Additionally, phenolic compounds in vinasses can inhibit seed germination and crop growth (Mattiazzo and de Glorie 1987).

Tequila and mezcal vinasses have similar physiochemical characteristics with sugarcane and beet vinasse, so similar effects in the soil could hope, for instance an unbalance of calcium, magnesium, potassium, and sodium content, that could increase the levels of these cations, organic carbon and phosphorous in the soil. Excess in the amount of vinasse irrigated increases the possible contamination of aquifers, detriments in crops yield and geobiochemical cycles functions. However, the level of these effects either positives or negatives will be influenced mainly by quantity of irrigation, composition of the vinasse, soil type, relief, crop type and season.

## Microbial populations affected by irrigation of vinasses

Soil microorganisms, such as fungi and bacteria, play a vital role in decomposition of organic material thereby releasing nutrients to soil (Yang et al. 2013). Christofoletti et al. (2013) found that the addition of sugarcane vinasse in the soil causes changes in the population of microorganisms in the soil, resulting in many alterations in the chemical processes, such as decomposition of the organic matter, nitrification, denitrification, fixation of air N_2_ and increase in pH. Little is known, however, how these microorganisms might be affected when vinasses are applied to soil. Results in the literature (only for sugarcane and sugar beet vinasses) are contrasting. For instance, Yang et al. (2013) reported that the addition of sugarcane vinasse to soil had little effect on the fungal population, but it increased the amount of Actinobacteria. This suggests changes in populations induced by vinasses compounds and conditions. Other authors have observed increase in different populations of microorganisms, for example dominance of fungi and bacteria (*Neurospora* ssp.*, Aspergillus* ssp.*, Penicillum* ssp.*, Mucor* ssp. and *Streptomyces* ssp.) (Camargo (1954) or alterations in actinomycetes and cellulolytic bacteria populations (Santos et al. 2009) in soils irrigated with sugarcane vinasse. Also, the application of sugarcane vinasses at high concentrations increased fungal growth (Santos et al. 2008), but inhibited growth of *Sclerotinia sclerotiorum, Pythium aphanidermatum* and *Phytophthora parasitica*, which are phytopathogens. Velásquez-Pomar and Sánchez de Prager (2011) reported a 70 % increase in external mycelium length, arbuscular mycorrhizal fungi and 10 % were more active when sugarcane vinasse were applied to soil compared to a treatment without vinasse. Studies with other residual water from distilleries reported that the addition of sugarcane vinasses to soil increased colony forming units (CFUs) significantly 25 % compared to soil without vinasses (Chaudhary et al. 2013). On the other hand, Tejada et al. (2007) reported that irrigated soil with sugar beet vinasse has a negative effect decreasing the microbial biomass, respiration and enzymatic activities due to affectation of enzyme such as dehydrogenase (related to oxidative phosphorylation processes), urease and BBA-protease (participation into N cycle), α-glucosidase (involved in the decomposition of plant remains), phosphatase (important to organic matter degradation) and arylsulfatase (hydrolysis of aromatic sulphate esters to phenols and sulphate) into the soil. All these enzymes are essential in the degradation and mineralization processes of organic material.

Inhibitory effects on growth of bacteria, such as *Geobacillus stearothermophylus, Staphylococus aureus, Escherichia coli, Bacillus cereus* and *Salmonella* spp., have been attributed to melanoidins present in vinasses (Arimi et al. 2014). These compounds inhibit enzymes that break down proteins (Ibarz et al. 2008) and are genotoxic by forming complex melanoidin-metals (e.g. Cu), which can affect DNA strands (Cämmerer et al. 2012). Other compounds in vinasse that might inhibit methanogens are polyphenols at 50–1000 mg L^−1^ (Arimi et al. 2014). This might inhibit COD removal during the treatment of vinasses before discharging them (Jiménez et al. 2003). In soil, inhibition of methanogens could alter the biogeochemical cycles. Arimi et al. (2014) conclude after extend review of sugarcane (molasses) vinasses that although polyphenols could have higher antimicrobial effects than melanoidin at the same concentration, melanoidins have the main antimicrobial effect as they are found at higher concentration (≈20 g L^−1^) than polyphenols.

All these reports indicate clearly that vinasses could affect positively or negatively the microbial populations in soil, as they contain large amounts of residual sugars, nutrients, melanoidins and polyphenols. However, it is not known how the soil microbial community structure changes, which group of organisms is favoured or inhibited or if exists succession of communities. So, more studies are needed to determine how different types of vinasses when applied to soil affect the microbial populations using molecular tools. Also in important remark that the little information in the literature is regarding sugarcane and beet vinasses, but not there is with mezcal or tequila.

## Greenhouse gases (GHG) emission due to vinasses application

The vinasses (sugarcane, sugar beet, mezcal and tequila) usually contain high amount of compounds phenol (34–542 mg L^−1^) (Table 2), high organic matter, carbohydrates, aromatic compounds, and other carbon-rich compounds. Probably, the microorganisms in soil used sugar and phenol compounds presents in irrigated vinasses producing CO_2_ emission. Few reports exist in the literature about GHG (CO_2_, CH_4_, N_2_O) with vinasses irrigation to soil. Although these effluents have a huge potential emission of GHG, not only when they are added to soil, in their storage, transportation or final disposal in rivers, lagoons or soils.

Goncalves de Oliveira et al. (2013) reported that the irrigation with sugarcane vinasse in agricultural soil tripled CO_2_ emissions under flooding conditions (200 m^3^ ha^−1^). Also these authors reported that the addition of sugarcane vinasses to soil generated fluxes of CH_4_ ranging from −64.4 to 3.1 and −42.0 to 44.3 μg m^−2^ h^−1^ for the control soil and the soil added with sugarcane vinasses, respectively. Additionally reported that the annual application of 46 kg of N ha^−1^ in form of sugarcane vinasse had a N_2_O emission of 0.31–0.52 kg ha^−1^. Other reports indicated increase of CO_2_, CH_4_ and N_2_O emission in field of sugarcane irrigated with alcohol vinasses with a total emission of 3000 kg CO_2_ equivalent ha^−1^ year^−1^ (Carmo et al. 2012).

The emission of N_2_O probably is due to the addition of vinasses and by both denitrification and nitrification path, depending on oxygenation conditions. Also, an increase of water content in the soil do that the denitrification became the dominant process for the N_2_O emission, due to the development of anaerobic sites that favour the reduction of nitrogen (Zhu et al. 2013). Also, in anaerobic conditions some microorganism can reduce nitrates and produce N_2_O, such as some arches for instance *Euryarchaeote* sp. (Hu et al. 2013).

On the other hand, the methane emission (CH_4_) is favoured with the anaerobic conditions and with high amount of organic matter in the soil (Yao et al. 1999). Soares et al. (2009) reported that an agricultural soil had not CH_4_ emissions significant after sugarcane vinasses irrigation, only when the soil was in anaerobic conditions for several hours.

So, application of vinasses to soil increase greenhouse gases (GHG) emissions. Moraes et al. (2014) stated that degradation of organic material in sugarcane vinasses when applied to soil increased GHG emissions. Thangarajan et al. (2013) reported that organic material applications contribute to higher GHG emissions, i.e. CO_2_, N_2_O and CH_4_. The GHG emissions from the application of different types of vinasses to soil was calculated using the equations given by Thangarajan et al. (2013) to estimate the potential emission of any waste and using an application rate of 200 m^3^ vinasse ha^−1^, which was suggested as optimal by Aparecida-Christofoletti et al. (2013) for sugarcane vinasses (Table 3).

**Table 3:** Estimations realized to greenhouse gas (GHG) emission for different kinds of vinasses and different systems or wastes

Kind of vinasses or system or waste	Vinasses generation L ×10^9 f^	Density Mg m^−3 h^	Mg of vinasses generated ×10^8^	Doses^g^ Ton ha^−1^	%C in vinasses	%N in vinasses	(CO_2_–C eq Gg year^−1^)		
							CO_2_	CH_4_	N_2_O
Sugarcane^a^	1400–2100	1.25^h^	18–26	382.5	0.29	0.123	92,015–138,022	1425–2138	13,169–19,753
Sugar beet^b^	200–320		2.5–3.9		0.52	0.475	13,245–20,604	368–572	7320–11,387
Mezcal^c^	0.0034–0.0063		0.00042–0.00079		N. R.	0.660	2–4	N.E	2–3
Tequila^d^	0.16–0.23		0.02–0.03		0.168	0.035	104–148	0.948–1.35	4–6
Continuous and rotation crops^e^	–	–	–	–	–	–	5.0–31	N.E	1.3×10^−4^
Rice crops^e^	–	–	–	–	–	–	0–29	N.E	1.1×10^−4^
Shrub land/natural landscape^e^	–	–	–	–	–	–	0.2–30	N.E	6.5×10^−3^
Animal waste^e^	–	–	–	–	–	–	4.1–4.7	N.E	0.05
Grazing^e^	–	–	–	–	–	–	1.7–28	N.E	3.7×10^−5^

^a^ Goldemberg et al. (2008); ^b^ Christodoulou and Bezergianni (2007); ^c^ CRM (2015); ^d^ CRT (2015); ^e^ Muñoz et al. (2010); *NR* not reported; *NE* not estimated. Equations to estimate the potential quantity of GHG (CO_2_, N_2_O and CH_4_) emissions: CO_2_–C (Gg year^−1^) = (((Potential quantity of vinasses produced (Mg year^−1^))/(potential application rate (t ha^−1^))) × C to CO_2_ emission factor (20 C t ha^−1^year^−1^)/1000; CH_4_–CO_2_–Ceq (Gg year^−1^) = potential quantity of vinasses produced (Mg year^−1^) × %C in vinasses × C to CH_4_ conversion (1.33) × 1/1000 × 21 × C to CH_4_ emission factor (1 %); N_2_O–CO_2_–Ceq (Gg year^−1^) = potential quantity of vinasses produced (Mg year^−1^) × %N in vinasses × N to N_2_O conversion (1.57) × 1/1000 × 310 × N to N_2_O emission factor (1.25 %); Where 1.33 is the C to CH_4_ conversion; 1 % is the C to CH_4_ emission factor; 21 and 310 are the global warming potential for CH_4_ and N_2_O according to (IPCC 2007); 1.57 is the N to N_2_O conversion; 1.25 % is the N to N_2_O emission factor; 1000 is the factor of conversions from Mg to Gg; ^f^ Estimated liters of vinasses generated in total production of different products; ^g^ Dose recommended by Aparecida-Christofoletti et al. (2013) 300 m^3^ ha^−1^ and converted to ton ha^−1^, ^h^ The density 1.25 Mg m^−3^ reported by Tejada et al. (2007) for beet vinasses was generalized to all vinasses analysed in this document and to estimate the GHG emissions

In general, agricultural systems contribute substantially to global fluxes of CO_2_, CH_4_ and N_2_O. Agriculture activities add 10–12 % to the total anthropogenic emissions of GHG, and organic material applications to soil are an important contributor to these emissions (IPCC 2007), i.e. rice crops, continuous and rotation crops. The calculations presented in this document (Table 3) indicated that sugarcane vinasses have the potential to generate between 5-4759 times more CO_2_ than that reported by rice crop (Muñoz et al. 2010) (Table 3). Goncalves de Oliveira et al. (2013) applied 200 m^3^ sugarcane vinasses ha^−1^ to soil generating emissions of 2387 and 1525 kg of CO_2_ eq ha^−1^ year^−1^ (N_2_O, CH_4_) when sugarcane was burned or left in the field, respectively. However, there are no reports on GHG emission when other vinasses are applied to soil. This suggests that further studies should be done to obtain data so that the contribution of vinasses to global warming can be calculated. However the data will be specific for each area, conditions, kind of soils, doses of irrigation, season, etc., but they can give more information of GHG contributions of this agricultural practice using any kind of vinasse.

## Perspectives

According to production data, the growth rate for alcohol from sugarcane production in the last five years was 19 %, for alcohol from beet 2 % (Gupta and Verma 2015), tequila and mezcal production increased 1 and 32 %, respectively (CRT 2015; CRM 2015). So, the annual average growth for vinasses production from these sources could be 3.6 % (equivalent to 7600 millions of liters of vinasse per year). The volume of vinasses produced by each industry together with the predicted annual increase represent both a huge technical and economical challenge if they want to be treated in wastewater treatment plants. Yet, they result in an issue with negative impact for the environment if these are disposed off without treatment or in high rate application to soil. Whether this issue is not attended in short time the consequences could be very negative to the environment.

Vinasses are classified as complex effluents and their treatment is not easy, therefore the agricultural soil irrigation has been the most common alternative for their final disposal. Some positive effects have been observed with the irrigation under certain conditions, but negative effects have been reported too. The fact is that vinasses irrigation could induce several changes, perturbation, or alterations in different compartments such as soil, water (rivers, lagoons, aquifers), air (GHG emissions), microorganisms, plants, etc., when they are irrigated/discharged at high rates. A complete solution is not foreseen in short time, so a regulation for soil application of the different kind of vinasses should be mandatory in all countries where they are generated. Also, recommended doses are necessary with the base of salt, phenols, organic material and cations (K^+^, Na^+^, Ca^++^, Mg^++^) content.

## Conclusions

When vinasses are applied to soil, soil fertility increases or decreases. Possible effects depend on type and application rates of vinasses, orographic characteristics, type of soil, chemical composition, crop type and climatic conditions. Addition of vinasses to soil increases EC, and the leaching of NO_3_^−^ and metals, such as zinc and copper, to aquifers. Consequently, possible negative effects of vinasse when applied to soil might be observed a long time after initial application.

The application of vinasses to soil can increase emissions of GHG (CH_4_, CO_2_ and N_2_O), as a result of their high organic matter content. An estimation of emissions of GHG when vinasses are applied to soil indicates a possible increase in fluxes of CH_4_, CO_2_ and N_2_O. However, field studies should be started so that experimental data are available to confirm these estimations. It is recommendable that environmental authorities establish regulations for the use of vinasses in agricultural systems and do not consider a single parameter, e.g. K_2_O content.

After all positive and negative effects pointed out about vinasses irrigation to soil, it seems clear that some suggestions could be applied in order to reduce the negative impacts to environment: (1) to decrease the doses of irrigation and avoid doing it in rainy season to prevent lixiviation of nutrients toward groundwater; (2) vinasses could be irrigated with previous dilution to adjust nutrients to requirements of specific crops and to avoid excesses of monovalent cations, which may cause leaching and/or nitrogen immobilization or toxicity to plants and to beneficial soil microorganisms; (3) aeration after irrigation with soil turning to avoid high GHG emissions generated by anaerobic conditions.

## References

[CR1] Algur OF, Kadioglu A (1992). The effects of vinasse on the growth, biomass, and primary productivity in pea (*Pisum sativum*) and sunflower (*Helianthus annuus*). Agric Ecosyst Environ.

[CR2] Aparecida-Christofoletti C, Pedro-Escher J, Evangelista-Correia J, Urbano-Marinho JF, Fontanetti CS (2013). Sugarcane vinasse: environmental implications of its use. Waste Manag.

[CR3] Arimi MM, Zhang Y, Götz G, Kiriamiti K, Geißen SU (2014). Antimicrobial colorants in molasses distillery wastewater and their removal technologies. Int Biodeterior Biodegrad.

[CR4] Bassanta MV, Dourado-Neto D, Reichardt K, Bacchi OOS, Oliveira JCM, Trivelin PCO, Timm LC, Tominaga TT, Correchel V, Cássaro FAM, Pires LF, de Macedo JR (2003). Management effects on nitrogen recovery in a sugarcane crop grown in Brazil. Geoderma.

[CR5] Bautista-Zúñiga F, Durán-de-Bazúa MdC (1998). Análisis del beneficio y riesgo potenciales de la aplicación al suelo de vinazas crudas y tratadas biológicamente. Rev Int Contam Ambient.

[CR6] Bengtsson G, Bengtson P, Mansson KF (2003). Gross nitrogen mineralization-, immobilization-, and nitrification rates as a function of soil C/N ratio and microbial activity. Soil Biol Biochem.

[CR7] Bermejo I (2010). Agricultura y cambio climático. El Ecologista.

[CR8] Brito LF, Marques J, Pereira JT, Souza ZM, La Scala N (2009). Soil CO_2_ emission of sugarcane field as affected by topography. Sci Agric.

[CR9] Buschiazzo DE, Estelrich HD, Aimar SB, Viglizzo E, Babinec FJ (2004). Soil texture and tree coverage influence on organic matter. Rangeland Ecol Manag.

[CR10] Camargo R (1954) O desenvolvimiento da flora microbiana nos solos tratados com vinhaca. PhD, Universidade de Sao Paulo

[CR11] Cämmerer B, Chodakowski K, Gienapp C, Wohak L, Hartwig A, Kroh L (2012). Pro-oxidative effects of melanoidinecopper complexes on isolated and cellular DNA. Eur Food Res Technol.

[CR12] Carmo JB, Filoso S, Zotelli LC, De Sousa Neto ER, Pitombo LM, Duarte-Neto PJ, Vargas VP, Andrade CA, Gava GJC, Rossetto R, Cantarella H, Neto AE, Martinelli LA (2012). Infield greenhouse gas emissions from sugarcane soils in Brazil: effects from synthetic and organic fertilizer application and crop trash accumulation. Glob Change Biol Bioenergy.

[CR13] Casa R, D’Annibale A, Pieruccetti F, Stazi SR, Giovannozzi-Sermanni G, Lo-Cascio B (2003). Reduction of the phenolic components in olive-mill wastewater by an enzymatic treatment and its impact on durum wheat (*Triticum durum Desf.*) germinability. Chemosphere.

[CR14] Cavalett O, Junqueira TL, Dias MOS, Jesus CDF, Mantelatto PE, Cunha MP (2012). Environmental and economic assessment of sugarcane first generation biorefineries in Brazil. Agric Ecosyst Environ.

[CR15] CETESB (2006) Vinhaça: critérios e procedimentos para aplicação no solo agrícola. Norma Técnica P4.231. São Paulo

[CR16] Chaudhary A, Sharma AK, Singh B (2013). Application of distillery effluent irrigation to agriculture soil and profiling of biochemical activity. Ann Plant Protect Sci.

[CR17] Christodoulou P, Bezergianni S (2007) The competitiveness of bioethanol production from sugar beet. Paper presented at the proceedings of general assembly meeting of international commission for sugar technology—CITS

[CR18] Christofoletti CA, Escher JP, Correia JE, Marinho JF, Fontanetti CS (2013). Sugarcane vinasse: environmental implications of its use. Waste Manag.

[CR06] Conde-Bueno P, Martín-Rubí JA, García-Giménez R, Jiménez-Ballesta R (2009) Impacts caused by the addition of wine vinasse on some chemical and mineralogical properties of a Luvisol and a Vertisol in La Mancha (Central Spain). J Soils Sediments 9:121–128

[CR19] CRM (2015) Consejo Regulador del Mezcal. Informes. http://www.crm.org.mx/. Accessed 16 January 2015

[CR20] CRT (2015) Consejo Regulador del Tequila. Información Estadística. http://www.crt.org.mx/EstadisticasCRTweb/. Accessed 16 January 2015

[CR21] da Silva A, Rossetto R, Bonnecine J, Piemonte M, Muraoka T (2012). Net and Potential Nitrogen Mineralization in Soil with Sugarcane Vinasse. Sugar Tech.

[CR22] EPA (2016) Resource Conservation and Recovery Act (RCRA) Regulations. http://www.epa.gov/rcra/resource-conservation-and-recovery-act-rcra-regulations#nonhaz. Accessed 02 March 2016

[CR90] España-Gamboa E, Mijangos-Cortes J, Barahona-Perez L, Dominguez-Maldonado J, Hernández-Zarate G, Alzate- Gaviria L (2011) Vinasses: characterization and treatments. Waste Manag Res 29:1235–1250 10.1177/0734242X10387313 21242176

[CR23] FAO (2000). Fertilezers and their use.

[CR24] FAO (2015) Statistical data warehouse. http://data.fao.org/es/statistics. Accessed December 2015

[CR25] Fuess LT, Garcia ML (2014). Implications of stillage land disposal: a critical review on the impacts of fertigation. J Environ Manag.

[CR26] Gemtos TA, Chouliaras N, Marakis S (1999). Vinasse rate, time of application and compaction e!ect on soil properties and durum wheat crop. J Agric Eng Res.

[CR07] Goldemberg J, Teixeira-Coelho S, Guardabassi P (2008) The sustainability of ethanol production from sugarcane. Energ Policy 36:2086–2097

[CR27] Goncalves de Oliveira B, Nunes-Carvalho JL, Pellegrino-Cerri CE, Clemente-Cerri C, Feigl BJ (2013). Soil greenhouse gas fluxes from vinasse application in Brazilian sugarcane areas. Geoderma.

[CR28] Gupta A, Verma JP (2015). Sustainable bio-ethanol production from agro-residues: a review. Renew Sustain Energy Rev.

[CR29] Hu H-W, Zhang L-M, Yuan C-L, He J-Z (2013). Contrasting Euryarchaeota communities between upland and paddy soils exhibited similar pH-impacted biogeographic patterns. Soil Biol Biochem.

[CR30] Ibarz A, Garza S, Pagán J (2008). Inhibitory effect of melanoidins from glucose–asparagine on carboxypeptidases activity. Eur Food Res Technol.

[CR31] Iñiguez G, Acosta TN, Martínez CL, Parra J, González Q (2005). Utilización de subproductos de la industria tequilera. Parte 7. Compostaje de bagazo de agave y vinazas tequileras. Rev Int Contam Ambient.

[CR32] IPCC (2007) Climate change 2007. The physical science basis: contribution of working group I to the IV assessment report of the intergovernmental panel on climate change. https://www.ipcc.ch/publications_and_data/publications_and_data_reports.shtml

[CR33] Jiménez AM, Borja R, Martín A (2003). Aerobic/anaerobic biodegradation of beet molasses alcoholic fermentation wastewater. Process Biochem.

[CR34] Joersbo M, Pau EC, Davey MR (2007). Sugar beet. Transgenic crops IV. Biotechnology in agriculture and forestry.

[CR35] Kabir Z, O’Halloran J, Hamel C (1998). Dynamics of the mycorrhizal symbiosis of corn (*Zea mays* L.): effects of host physiology, tillage practice and fertilization on spatial distribution of extraradical mycorrhizal hyphae in the field. Agric Ecosyst Environ.

[CR36] Laime EMO, Fernandes PD, Oliveira DCS, Freire EA (2011). Possibilidades tecnológicas para a destinação da vinhaça: uma revisão. Revista Trópica – Ciências Agrárias e Biológicas.

[CR37] López-López A, Davila-Vazquez G, León-Becerril E, Villegas-García E, Gallardo-Valdez J (2010). Tequila vinasses: generation and full scale treatment processes. Rev Environ Sci Biotechnol.

[CR38] Madejón E, López R, Murillo JM, Cabrera F (2001). Agricultural use of three (sugarbeet) vinasse composts: effect on crops and chemical properties of a Cambisol soil in the Guadalquivir river valley (SW Spain). Agric Ecosyst Environ.

[CR39] Madrid L, Díaz-Barrientos E (1998). Release of metals from homogenous soil columns by wastewater from an agricultural industry. Environ Pollut.

[CR40] Martín-Olmedo P, Murillo JM, Cabrera F, López R (1999). Sugarbeet (*Beta vulgaris*) response to residual soil N under Mediterranean agronomic practices. J Agric Sci.

[CR41] Mattiazzo ME, de Glorie NA (1987). Effect of vinasse on soil acidity. Water Sci Technol.

[CR42] Mavi MS, Sanderman J, Chittleborough DJ, Cox JW, Marschner P (2012). Sorption of dissolved organic matter in salt-affected soils: effect of salinity, sodicity and texture. Sci Total Environ.

[CR43] Méndez-Acosta HO, Snell-Castro R, Alcaraz-González V, González-Álvarez V, Pelayo-Ortiz C (2010). Anaerobic treatment of Tequila vinasses in a CSTR-type digester. Biodegradation.

[CR44] Moraes SB, Junqueira TL, Pavanello LG, Cavalett O, Mantelatto PE, Bonomi A, Zaiat M (2014). Anaerobic digestion of vinasse from sugarcane biorefineries in Brazil from energy, environmental, and economic perspectives: Profit or expense?. Appl Energy.

[CR45] Muñoz C, Paulino L, Monreal C, Zagal E (2010). Greenhause gas (CO_2_ and N_2_O) emissions from soils: a review. Chil J Agric Res.

[CR04] Núñez-Zofío M, Larregla S, Garbisu C, Guerrero MM, Lacasa CM, Lacasa A (2013) Application of sugar beet vinasse followed by solarization reduces the incidence of Meloidogyne incognita in pepper crops while improving soil quality. Phytoparasitica 41:181–191

[CR46] Ortegon GP, Arboleda FM, Candela L, Tamoh K, Valdes-Abellan J (2016). Vinasse application to sugar cane fields. Effect on the unsaturated zone and groundwater at Valle del Cauca (Colombia). Sci Total Environ.

[CR47] Osman KT (2013). Soils: principles, properties and management.

[CR48] Pant D, Adholeya A (2007). Biological approaches for treatment of distillery wastewater: a review. Bioresour Technol.

[CR49] Parnaudeau V, Condom N, Oliver R, Cazevieille P, Recous S (2008). Vinasse organic matter quality and mineralization potential, as influenced by raw material, fermentation and concentration processes. Bioresour Technol.

[CR50] Poz-Gonzalo DD, Casagrande J, Soares M, Mouta E (2006) Effect of High Levels of Vinasse Application on Soil Fertility and Potash Leaching. Paper presented at the 18th World Congress of Soil Science, Philadelphia, Pennsylvania, USA, Friday, 14 July 2006

[CR51] Prasad KR, Kumar RR, Srivastava SN (2008). Design of optimum response surface experiments for electro-coagulation of distillery spent wash. Water Air Soil Pollut.

[CR52] Qiu S, Xie J, Zhao S, Xu X, Hou Y, Wang X, Zhou W, He P, Johnston AM, Christie P, Jin J (2014). Long-term effects of potassium fertilization on yield, efficiency, and soil fertility status in a rain-fed maize system in northeast China. Field Crops Res.

[CR53] Ribeiro BT, Lima JM, Guilherme LRG, Julião LGF (2010). Lead sorption and leaching from an Inceptisol sample amended with sugarcane vinasse. Sci Agric.

[CR54] Robles-González V, Galíndez-Mayer J, Rinderknecht-Seijas N, Poggi-Varaldo H (2012). Treatment of mezcal vinasses: a review. J Biotechnol.

[CR55] Roy RN, Frinck A, Blair GJ, Tandon HLS, FAO (2006). Nutrient management guidelines for some major field crops. Plant nutrition for food security. A guide for integrated nutrient management.

[CR56] Santos M, Diánez F, de Cara M, Tello JC (2008). Possibilities of the use of vinasses in the control of fungi phutopathogens. Bioresour Technol.

[CR01] Santos TMC, Santos MAL, Santos CG, Santos VR (2009) Efeito da fertirrigação com vinhaça nos microrganismos do solo. Rev Caatinga 22:155–160

[CR02] Santos C, Lucas MS, Dias AA, Bezerra RMF, Peres JA, Sampaio A (2014) Winery wastewater treatment by combination of Cryptococcus laurentii and Fenton’s reagent. Chemosphere 117:53–58 10.1016/j.chemosphere.2014.05.083 24968162

[CR57] Soares LHB, Alves BJR, Urquiaga S, Boddey RM (2009). Mitigação das emissões de gases efeito estufa pelo uso de etanol da cana-de-açúcar produzido no Brasil. Circular Técnica.

[CR58] SSSA (2016) Eutrophication. www.soils.org. Accessed 02 March 2016

[CR59] Tejada M, Gonzalez JL (2006). The relationships between erodibility and erosion in a soil treated with two organic amendments. Soil Tillage Res.

[CR60] Tejada M, Moreno JL, Hernandez MT, Garcia C (2007). Application of two beet vinasse forms in soil restoration: Effects on soil properties in and environment in southern Spain. Agric Ecosyst Environ.

[CR61] Tejada M, García-Martínez AM, Parrado J (2009). Effects of a vermicompost composted with beet vinasse on soil properties, soil losses and soil restoration. Catena.

[CR62] Thangarajan R, Bolan NS, Tian G, Naidu R, Kunhikrishnan A (2013). Role of organic amendment application on greenhouse gas emission from soil. Sci Total Environ.

[CR91] USDA (2014) Soil Survey Manual. United States Department of Agriculture, Washington, DC

[CR63] USEPA (2004) Guidelines for Water Reuse. EPA/625/R-04/108 Washington, DC

[CR64] Velásquez-Pomar DC, Sánchez de Prager M (2011). Efecto de Vinazas sobre Hongos que Forman Micorriza Arbuscular en un Molisol del Valle del Cauca, Colombia. Rev Fac Nac Agron.

[CR65] Vlyssides A, Barampouti EM, Mai S, Stamatoglou A, Tsimas E (2010). Alternative biological systems for the treatment of vinasse from wine. Water Sci Technol.

[CR03] Wrage N, Velthof GL, van Beusichem ML, Oenema O (2001) Role of nitrifier denitrification in the production of nitrous oxide. Soil Biol Biochem 33:1723–1732

[CR66] Xu P, Liang LZ, Dong XY, Xu J, Jiang PK, Shen RF (2014). Response of Soil Phosphorus Required for Maximum Growth of *Asparagus officinalis* L. to Inoculation of Arbuscular Mycorrhizal Fungi. Pedosphere.

[CR67] Yang SD, Liu JX, Wu J, Tan HW, Li YR (2013). Effects of Vinasse and Press Mud Application on the Biological Properties of Soils and Productivity of Sugarcane. Sugar Tech.

[CR68] Yao H, Conrad R, Wassmann R, Neue HU (1999). Effect of soil characteristics on sequential reduction and methane production in sixteen rice paddy soils from China, the Philippines, and Italy. Biogeochemistry.

[CR69] Yavuz Y (2007). EC and EF processes for the treatment of alcohol distillery wastewater. Sep Purif Technol.

[CR70] Zhu T, Zhang J, Yang W, Cai Z (2013). Effects of organic material amendment and water content on NO, N_2_O, and N_2_ emissions in a nitrate-rich vegetable soil. Biol Fert Soils.

